# Proteoglycan-4 is correlated with longer survival in HCC patients and enhances sorafenib and regorafenib effectiveness via CD44 in vitro

**DOI:** 10.1038/s41419-020-03180-8

**Published:** 2020-11-16

**Authors:** Francesco Dituri, Rosanna Scialpi, Tannin A. Schmidt, Martina Frusciante, Serena Mancarella, Luigi Giovanni Lupo, Erica Villa, Gianluigi Giannelli

**Affiliations:** 1 National Institute of Gastroenterology “S. De Bellis” Research Hospital, 70013 Castellana Grotte, Italy; 2 Biomedical Engineering Department, University of Connecticut Health Centre, Farmington, CT USA; 3 University of Bari, Department of General Surgery and Liver Transplantation, Policlinico - piazza Giulio Cesare 14, 70125 Bari, Italy; 4 Gastroenterology Unit, Department of Internal Medicine, University of Modena and Reggio Emilia, Modena, Italy

**Keywords:** Targeted therapies, Preclinical research

## Abstract

Sorafenib and regorafenib administration is among the preferential approaches to treat hepatocellular carcinoma (HCC), but does not provide satisfactory benefits. Intensive crosstalk occurring between cancer cells and other multiple non-cancerous cell subsets present in the surrounding microenvironment is assumed to affect tumor progression. This interplay is mediated by a number of soluble and structural extracellular matrix (ECM) proteins enriching the stromal milieu. Here we assess the HCC tumor expression of the ECM protein proteoglycan 4 (PRG4) and its potential pharmacologic activity either alone, or in combination with sorafenib and regorafenib. PRG4 mRNA levels resulted strongly correlated with increased survival rate of HCC patients (*p* = 0.000) in a prospective study involving 78 HCC subjects. We next showed that transforming growth factor beta stimulates PRG4 expression and secretion by primary human HCC cancer-associated fibroblasts, non-invasive HCC cell lines, and ex vivo specimens. By functional tests we found that recombinant human PRG4 (rhPRG4) impairs HCC cell migration. More importantly, the treatment of HCC cells expressing CD44 (the main PRG4 receptor) with rhPRG4 dramatically enhances the growth-limiting capacity of sorafenib and regorafenib, whereas not significantly affecting cell proliferation per se. Conversely, rhPRG4 only poorly potentiates drug effectiveness on low CD44-expressing or stably CD44-silenced HCC cells. Overall, these data suggest that the physiologically-produced compound PRG4 may function as a novel tumor-suppressive agent by strengthening sorafenib and regorafenib effects in the treatment of HCC.

## Introduction

Hepatocellular carcinoma (HCC) is among the most frequent causes of cancer-related death worldwide. As the majority of patients with HCC is not eligible for curative treatments based on surgical approaches or radiofrequency ablation, systemic drug-based therapies have to be implemented. However, multi-year experiences of the administration of compounds such as sorafenib and regorafenib have yielded disappointing results in terms of overall survival^1–4^. A poor knowledge of the molecular mechanisms underlying HCC tumor progression, as well as the high inter- and intra-tumor heterogeneity exhibited by this neoplasm owing to concomitant preexisting liver disease, hamper the development of a precision medicine approach^5–7^. The crosstalk between the epithelial cancer cells and the surrounding microenvironment, in particular the cancer-associated fibroblasts (CAFs), is believed to play a key role in HCC progression and consequently exerts a critical influence on the clinical outcome^8,9^.

The origin of CAFs is still a matter of debate. Diverse intrahepatic cell types are suggested to undergo phenotypical changes, ultimately differentiating into CAFs during the inflammatory and progressive fibrogenic evolution of chronic liver disease, as well as during HCC development. In this context, CAFs may be phenotypically programmed by adjacent malignant cells and, in turn, enhance HCC cells proliferation and spread, likely through the deposition or secretion of different molecules including extracellular matrix (ECM) proteins^10,11^. Indeed, CAFs are the major contributors of ECM deposition, including fibrillar collagen type I and III, and non-collagenous glycoproteins, such as fibronectin (FN), laminin, hyaluronan, elastin, and proteoglycans (PGs)^12^. PGs are a class of heavily glycosylated high molecular weight proteins, which are widely expressed in all fibrotic tissues, in particular in the cartilage tissues, where they have a lubrication function, allowing the sliding of the joints. A number of studies attributed ambiguous roles to certain PGs in cancer progression. Soluble biglycan can interact with different combinations of multiple surface receptors, including toll-like receptors (TLRs) 2 and 4, CD14 and CD44, that in turn influence processes such as autophagy, angiogenesis, cell growth and migration, to finally promote tumor progression or suppression. Hyaluronan and versican (VCAN), via binding the same receptors, were found to promote tumor cell proliferation and metastatic ability^13–15^. Some PGs, such as biglycan and VCAN, are upregulated by transforming growth factor (TGF)-β^15,16^. TGFβ has been widely reported to promote a more invasive and aggressive phenotype in HCC^11,17–20^. A TGFβ receptor 1 inhibitor, galunisertib, proved effective in a multicentric clinical trial in patients with advanced HCC^21^. Furthermore, we have also shown that in preclinical experimental models, inhibiting TGFβ signaling decreased HCC aggressiveness, reducing CD44 expression^22^. Recently, it was demonstrated that the human recombinant form of the proteoglycan 4 (PRG4) inhibits TGFβ-mediated invasiveness of breast cancer cells by binding to CD44 and affecting its downstream signaling pathway^23^.

CD44 was already well-established as a widely distributed receptor for diverse ligands, including hyaluronan, osteopontin, and matrix metalloproteinases^24^. In light of recently accumulated knowledge, it is increasingly regarded as a stemness marker in HCC^22^. The overexpression of CD44 in this cancer is an early event during carcinogenesis initiation, which is responsible for the acquisition of a senescence-resistant phenotype and the accumulation of mutations by hepatocytes undergoing transformation^25^. In preclinical HCC experimental models, CD44 expression was shown to be increased by TGFβ and countered by galunisertib, consistent with the efficacy reported for this drug in the multicentric clinical trial in HCC patients^21,22,26^. Furthermore, both in in vitro and in vivo settings, CD44 expression was reported to promote the acquisition of sorafenib resistance by liver cancer cells once they had gained a mesenchymal-like transformation status as a result of persistent exposure to TGFβ^27^.

The expression of PRG4 was primarily detected in synovial fluid, where it contributes to boundary lubrication of synovial joints^28–31^. Synovial fibroblasts and chondrocytes are a main source of this PG^29,32^. Interestingly, PRG4 concentrations resulted significantly reduced in synovial fluid of patients with chronic osteoarthritis, and restoration of joints lubrication could be obtained following intra-articular PRG4 supplementation in these subjects^33^. The evidence that this PG may act to attenuate friction-related joint degeneration is further corroborated by studies using different mouse models of osteoarthritis, in which disease progression could be prevented by PRG4 overexpression^34,35^.

In light of the potential antitumor function of PRG4, and the relevant involvement of its recently identified receptor, CD44, in HCC progression, we aimed to investigate the role of this PG in a patients setting, as well as test its capacity to limit the aggressive phenotype of HCC cells and enhance the in vitro cell growth-inhibitory potential of sorafenib and regorafenib.

## Materials and methods

### Cells and reagents

HLE and HLF cell lines were purchased from JCRB Cell Bank (Japan). Hep3B and PLC/PRF/5 cell lines from ATCC (USA). All these cell lines were cultured in DMEM (Dulbeccoo’s Modified Eagle Medium) supplemented with sodium pyruvate, antibiotic–antimycotic, Hepes, and 10% fetal bovine serum (FBS) (Thermo Fisher Scientific). Cells were tested for the absence of mycoplasma contamination using the MycoFluor™ Mycoplasma Detection Kit (Thermo Fisher Scientific). Full-length recombinant human PRG4 (rhPRG4) was provided by Lubris Biopharma (Weston, MA, USA). Galunisertib (LY2157299), sorafenib (BAY 43-9006), and regorafenib (BAY 73-4506) were purchased from Cayman Chemicals (Ann Arbor, MI, USA). Antibodies used are listed in Supplementary Table 1.

### Western blot

Tissue proteins were extracted using T-PER Tissue Protein Extraction Reagent Supplemented with Halt Protease and Phosphatase Inhibitor Cocktail EDTA-free (Thermo Fisher Scientific). In brief, proteins were extracted using a tissue homogenizer. The lysates were incubated on ice for 30 min and vortexed every 10 min. Then, the samples were clarified through centrifugation at 13,000 rpm (at 4 °C) for 20 min to precipitate insoluble debris. The supernatants (containing the extracted proteins) were assayed for protein concentration using Bradford Reagent (Bio-Rad). The proteins were then mixed with Laemmli buffer and 10% β-mercapto ethanol (BME), and denatured at 95 °C for 5 min. Ten to 20 µg of total proteins were loaded onto 4–20% polyacrylamide gradient gels and run in sodium dodecyl sulfate polyacrylamide gel electrophoresis. After separation, the proteins were transferred onto nitrocellulose membrane (Trans-Blot Turbo Mini 0.2 µm Nitrocellulose Transfer Packs, Bio-Rad) using the Trans-Blot Turbo Transfer System (Bio-Rad), stained with primary and horseradish peroxidase-conjugated secondary antibodies, and revealed using the Clarity Max Western ECL Substrate (Bio-Rad).

### Ex vivo HCC tissues isolation and treatment

Immediately after surgical resection, freshly collected HCC tumor specimens (~1 cm in size) were preserved in MACS Tissue Storage Solution (Miltenyi Biotec), further cut into smaller pieces (a few mm in size), and washed twice with Iscove’s Modified Dulbecco’s Media (IMDM) serum-free medium. Multiple (≥9) tissue pieces from random areas of tumors were cultured in IMDM serum free containing dimethyl sulfoxide (DMSO), LY2157299, TGFβ1 + DMSO, or LY2157299 + TGFβ1 under normal cell culture conditions (37 °C, 5% CO_2_). Culture medium and treatments were renewed after 24 hours. At the 48-hour end point the tissue specimens were washed twice with ice-cold PBS, snap-frozen in liquid nitrogen and finally stored at −80 °C.

### RNA extraction and cDNA synthesis

Thirty to 60 µg of frozen ex vivo treated HCC tissues were ground with a mortar-pestle in the presence of liquid nitrogen until a thin powder was obtained. The ground tissues were lysed with 0.5–1 ml of RLT buffer + 1% BME and then processed according to the manufacturer’s recommendations (RNeasy kit, Qiagen). RNA isolation from CAFs was performed following the procedure suggested by the RNeasy kit handbook. The obtained RNA was assayed for quality and concentration using the NanoDrop 2000/2000c (Thermo Fisher Scientific). cDNA was synthesized using the High Capacity cDNA reverse transcription kit (Thermo Fisher Scientific), according to the relative datasheet.

### Real-time polymerase chain reaction (qPCR)

One ng/µl of cDNA was used in 20 µl total reaction mix in the presence of 500 nM of each forward and reverse primer referred to a specific gene of interest, and 2× SYBR green master mix (Bio-Rad). The reaction was conducted in a CFX96 Touch Real-Time Detection System (Bio-Rad). The sequences of primers used are listed in Supplementary Table 2.

### Immunofluorescence

A cryostat microtome was used to cut HCC tumor samples into 5 µm thick slices. Slices were incubated with blocking buffer (10% FBS in Roswell Park Memorial Institute Medium) for 30 min to minimize nonspecific antibody binding, then incubated for 2 hours with primary antibodies diluted in the same buffer, washed three times with PBS (each wash for 5 min under shaking) and finally incubated with secondary AF488- or AF594-conjugated antibodies. At the end of this step the slices were washed four times as previously described, and mounted with 4′,6-diamidino-2-phenylindole-supplemented Vectashield anti-fade mounting medium.

### Stable CD44 silencing

HLE and HLF cell lines were transduced with lentiviral particles carrying control non-targeting (V), or specific CD44-targeting shRNA sequences (A to D), and selected with puromycin dihydrochloride (Thermo Fisher Scientific) to obtain stable CD44 silencing, according to the manufacturer’s instructions (OriGene Technologies, Inc., Rockville, MD 20850, USA). Control-shRNA sequence (V) and CD44-shRNA sequence B were used in all the experiments involving CD44 downregulation. CD44-silencing efficiency is shown in Supplementary Fig. 1.

### Proliferation assay

The assay was performed as previously described^36^. In brief, in 100 µl complete medium, 3000 cells were seeded in wells of a 96-well plate and left overnight to allow complete attachment. The next day (at time *t* = 0 hours), the medium was removed and replaced with 100 µl of fresh medium in the presence/absence of sorafenib (2.5 µM), regorafenib (2.5 µM), rhPRG4 (12.5–100 µg/ml). DMSO and PBS + 0.01% Tween-20 were used as vehicles of sorafenib/regorafenib, and rhPRG4, respectively. Cells in triplicate wells for each cell line were fixed at *t* = 0 hours with 4% paraformaldehyde (PFA, pH 7.6, 10 min incubation), then stained with crystal violet (CV) and thoroughly washed to remove excess staining. After 72 hours, the cells were fixed by adding 100 µl of 4% PFA directly to the medium (2% final PFA concentration, 20 min incubation), and processed for CV staining as previously described. Then, 100 or 200 µl of 1% sodium dodecyl sulfate were added to the wells and the plates were left under shaking until complete CV release from stained cells. Optical density was read at 595 nm wavelength, measured using a plate reader.

### Adhesion assay

The assay was performed as previously described^17^. In brief, 50,000 cells were diluted in 100 µl of serum-free DMEM medium (+0.5% BSA) and seeded onto uncoated, rhPRG4, or FN-coated wells of a 96-well plate, and then incubated at 37 °C, 5% CO2 for 30 minutes. An equal volume of 4% PFA (pH 7.2 in PBS) was added and the plates were immediately flicked for a few seconds to allow mixing. Thirty minutes later, the medium was removed and the adherent cells were stained with CV for 10 min. After abundant washing with tap water and distilled water the stained cells were allowed to dry out and, the next day, solubilized with 100 µl of 1% sodium dodecyl sulfate in water. Absorbance was read at 595 nm and proportionally related to the number of adhered cells.

### Transwell migration assay

The assay was performed as previously described^17^. In brief, 15,000 cells were suspended in 200 µl of serum-free DMEM medium containing 0.5% bovine serum albumin (BSA) loaded onto the top chamber of the transwell, whose membrane had previously been coated with FN on the lower surface, and left to migrate for 16 hours in the presence/absence of rhPRG4 (25 µg/ml) diluted in serum-free DMEM medium + 0.5% BSA in the lower chamber. The cells were then paraformaldehyde-fixed and stained with CV. Five fields/membrane were captured and the number of cells/field was measured.

### CAFs isolation

CAFs were isolated as previously described^10^. In brief, shortly after surgical resection, HCC tumor and peritumor specimens were minced into 0.5–1 cm pieces and left in MACS Tissue Storage Solution (Miltenyi Biotec). The tissues were then further cut into smaller size pieces (1–2 mm), washed three times in Hanks balanced salt solution (HBSS), and then incubated in HBSS in the presence of type IV collagenase (Thermo Fisher Scientific) and 3 mM CaCl2 at 37 °C under gentle rotation for 4 hours. At the end of this step, the dissociation was mechanically facilitated by pipetting up–down the digested tissues with a large size orifice 50 ml pipette. The floating cells were collected and washed three times with HBSS and seeded in normal culture conditions in IMDM + 20% FBS. The decanted, partially digested tissue specimens were subjected to a second round of collagenase digestion. The resulting dissociated cells were washed with HBSS and cultured in IMDM + 20% FBS. These cells underwent few (<5) passages to allow the loss of epithelial/immune/non adherent cells. To assure the purity of CAFs preparations, immunofluorescence or flow cytometry analyses were performed to evaluate the expression of mesenchymal markers (vimentin, αSMA, CD90). Contaminating non-fibroblastic cells (mostly cancerous hepatocytes, cholangiocytes, or macrophages), when present, were minimal, and evaluated using antibodies to EpCAM, CD133, CD45, OV6, CK19, and CD11b (Supplementary Fig. 2).

### CAFs treatments and PRG4 immunodepletion of CAFs-conditioned medium

CAFs were treated for 48 hours in the presence/absence of TGFβ1 (Peprotech) at the final concentration of 5 ng/ml in complete IMDM medium (+20% FBS), then washed three times with serum-free medium, and incubated in serum-free medium for another 48 hours for secretome enrichment. The conditioned medium (CM) was then collected, concentrated using a centricon device (3 kDa cutoff, Merck-Millipore), and incubated with anti-PRG4, or isotype antibody (anti-pY397 FAK)-bound PBS pre-washed magnetic microbeads, according to the manufacturer’s instructions (SureBeads Protein B, Bio-Rad). The PRG4-depleted or non-depleted (control) conditioned CAFs medium were then assayed for protein concentration, and used for further tests.

### Isolation and characterization of the primary HCC cell line HLC19

The primary HCC cell line, HLC19, was isolated from freshly collected surgically resected HCC specimen following the same isolation procedure to isolate CAFs. The immunophenotypic characterization of cells was carried out after several (>10) culture passages, by using antibodies to detect stemness markers (OV6, CD133, CD44, and CD90), epithelial markers (AFP, E-Cadh, EpCAM), mesenchymal markers (Vim, N-Cadh, αSMA), and other cancer-related surface proteins (CD13, CD151) (Supplementary Fig. 3).

### Flow cytometry

Cells were detached by using trypsin, then resuspended in PBS + 0.5% BSA + 0.1% sodium azide and incubated on ice in the presence of appropriate antibodies for membrane antigens staining. Alternatively, the detached cells were fixed and permeabilized using the Foxp3/Transcription Factor Staining Buffer Set (eBioscience—Thermo Fisher Scientific), and then stained with the appropriate antibodies. After three washes the cells were finally resuspended in PBS + 0.1% sodium azide and analyzed using the Navios flow cytometer (Beckman Coulter).

### Statistical analysis

The Kaplan–Meier method was used to estimate the cumulative probability of overall survival of HCC patients. Patients were censored at the time of LT, death, or last available follow-up. Differences in observed probability were assessed using the log-rank test. *T* test (one tail, paired, or two tails, unpaired) was used for statistical analysis of qPCR and in vitro cell culture experiments.

### Ethics approval

This work was approved by the Local ethics committee, Azienda Ospedaliero Universitaria Consorziale Policlinico di Bari (Bari, Italy); protocol number: 254; date of release: February 2012.

## Results

### PRG4 expression in HCC tumor tissues is positively correlated with overall survival

**Fig. 1:**
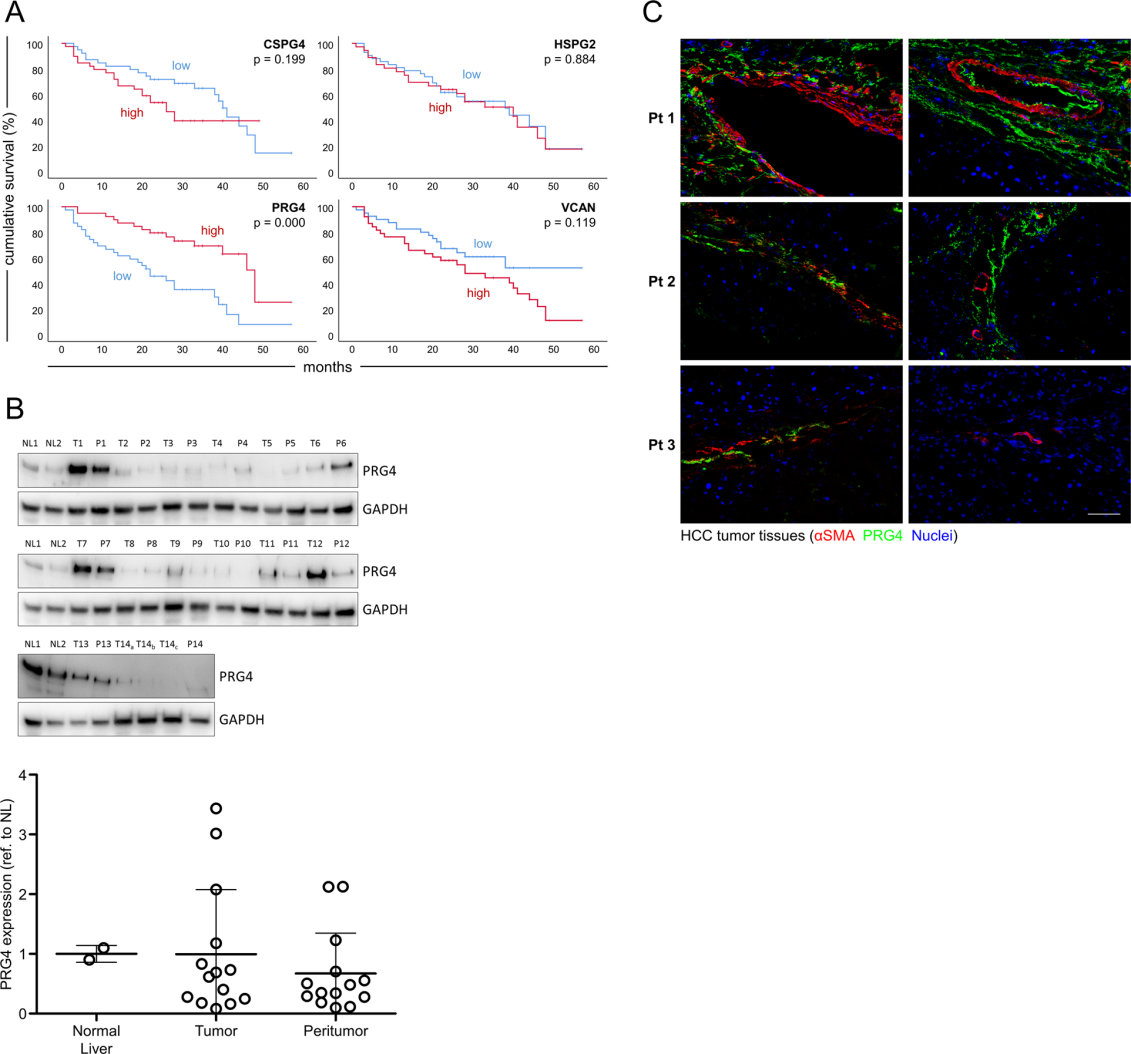
**PRG4 expression is positively correlated with HCC patients’ survival rates, and preferentially localized in the tumor stroma**. **a** Cumulative overall survival curves of HCC patients. Kaplan–Meier curves refer to patients stratified according to values of tumor mRNA expression indexes of PRG4 and three other main high molecular weight proteoglycans above or below the median. Tumor mRNA expression indexes of any specific marker used for generation of Kaplan–Meier curves represent the net tumor expression scores obtained by subtracting the non-tumor mRNA expression value from the corresponding value in matched tumor tissue in each patient. **b** Western blot analysis and quantification of PRG4 protein levels in tumor and peritumor paired tissues of 14 HCC patients; NL = normal liver; T = tumor tissue; P = peritumor tissue; T14_a_, T14_b_, and T14_c_ refer to three different areas of the same primary tumor nodule. GAPDH is used as housekeeping loading control. Densitometry analysis of bands was performed using ImageJ software. The signals of PRG4 bands were first normalized to those of GAPDH. The obtained normalized data were further normalized to the mean value of NL1 and NL2 (N1 and NL2 samples were loaded in all three membranes). The mean value of NL1 and NL2 in the graph is = 1 by definition. Data are the means ± SD. Whole blot scans are shown in Supplementary Fig. 5A. **c** Immunofluorescence of HCC tumor tissues (from three patients, Pt 1–3) displaying the localization of PRG4 and αSMA. Scale bar: 100 µm.

Through microarray gene expression analysis (data set: www.ncbi.nlm.nih.gov/geo; accession number: GSE54236), in a prospective study involving a cohort of 78 HCC patients we determined the mRNA expression levels of different PGs, including chondroitin sulfate proteoglycan 4 (CSPG4), perlecan (HSPG2), VCAN, and PRG4 in tumor and paired peritumor tissues. mRNA values of any PG gene in non-tumor tissues were subtracted from the values in the counterpart paired tumor specimens to obtain non-tumor-normalized net tumor gene expression indexes. The patients were stratified according to levels of these indexes above or below the median values. Remarkably, patients with higher expression levels of PRG4 survived longer, as shown by Kaplan–Meier analysis, as compared with those with lower expression levels (*p* = 0.000), whereas mRNA levels of CSPG4, HSPG2, and VCAN were not significantly correlated with survival (Fig. 1a). In conclusion, for the first time, herein we report that PRG4 is expressed in HCC tissues, and higher tumor levels of this PG are correlated with extended survival. To further confirm that PRG4 is expressed in liver of HCC patients we assessed by western blot the presence of PRG4 protein in two liver specimens from non hepatopathic subjects and 14 tumor specimens, along with paired surrounding non-cancerous specimens, from these 14 HCC patients (Fig. 1b, upper). After densitometry quantification, we determined that the protein expression levels of PRG4 are similar among normal liver, tumor, and peritumor tissues, although highly variable among different samples (Fig. 1b, lower). This suggests that the amount of PRG4 protein expression is not an epiphenomenon related to tumor development but rather a unique feature of individual tumor or peritumor tissue microenvironments. To evaluate the tissue sub-localization of PRG4 in HCC tumors, we visualized the expression of this PG by immunofluorescence (Fig. 1c). PRG4 results mainly distributed in the stromal compartment of the tumor, being mostly detectable in the proximity of alpha smooth muscle actin positive (αSMA+) cells. Less frequently, PRG4 is localized in αSMA− areas, which we assume to be populated by parenchymal or less “reactive” stromal cells. This suggests that PRG4 is predominantly produced in areas of intense myofibroblast secretory activity.

### TGFβ stimulation increases PRG4 expression in HCC ex vivo tissues, CAFs, and cancer cells

**Fig. 2:**
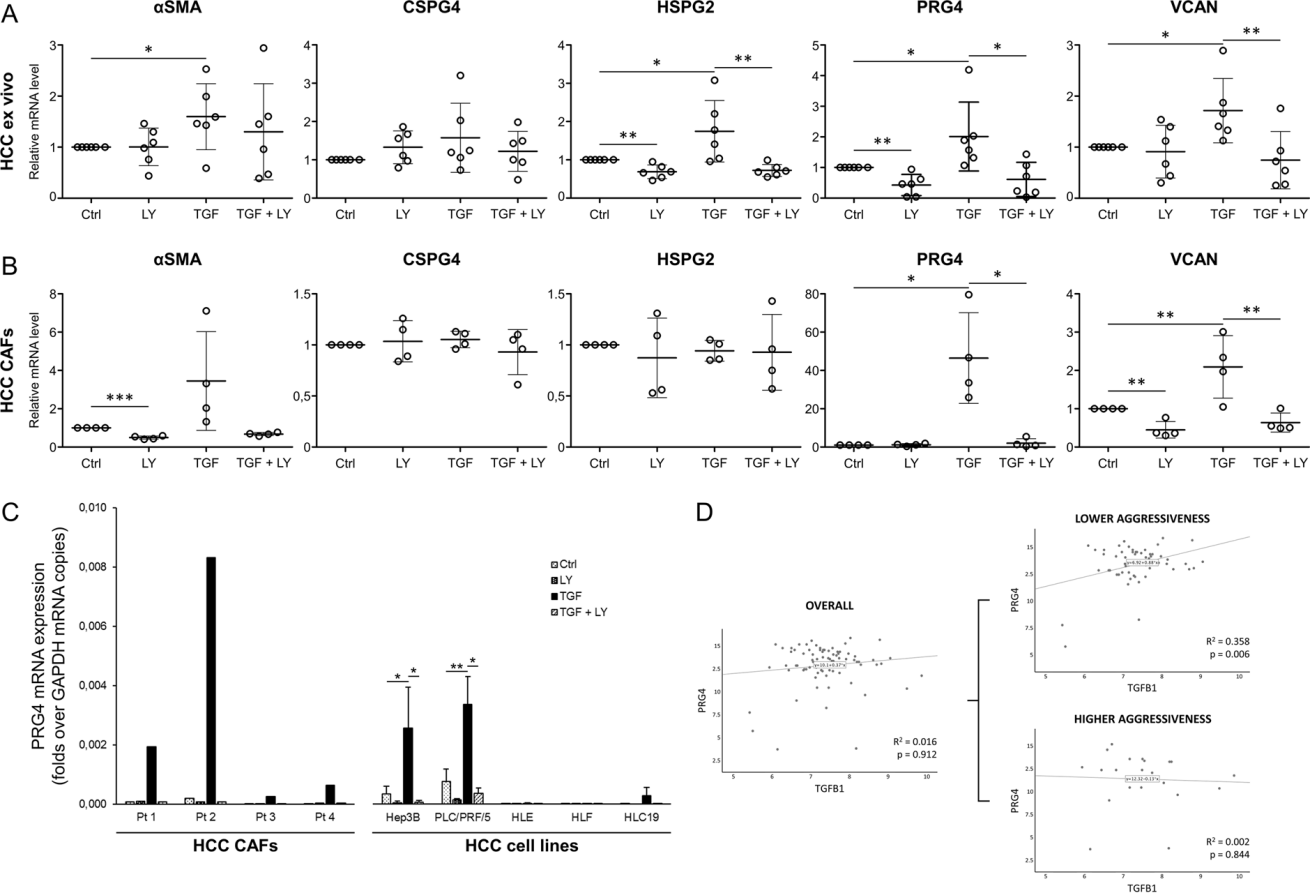
**TGFβ induces PRG4 expression in ex vivo cultured patients-derived HCC tumor samples, HCC CAFs, and cancer cells**. HCC patient-derived tumor specimens, CAFs and cancer cell lines were incubated for 48 hours in serum-free condition in the presence of DMSO, LY2157299 (LY, 10 µM), TGFβ1 (TGF, 5 ng/ml) + DMSO (diluted to 1:1000), or TGF + LY. Medium was replaced and treatments renewed at the 24 hours’ intermediate time point. mRNA expression levels of αSMA, PRG4, and other PGs were evaluated by qPCR. **a** Effect of TGFβ and/or LY2157299 on mRNA expression of αSMA, PRG4, and other PGs in ex vivo cultured patients-derived HCC tissues (*N* = 6 patients). Changes in αSMA mRNA expression were used as TGFβ response readout. **b** Effect of TGFβ and/or LY2157299 on mRNA expression of αSMA, PRG4, and other PGs in patients-derived HCC CAFs (*N* = 4 patients). Changes in αSMA mRNA expression were used as TGFβ response readout. **c** Comparison between raw levels of PRG4 mRNA expression of CAFs from four HCC patients (Pt 1–4) and five HCC cell lines in both basal condition, and in response to TGFβ and/or LY2157299 treatment. Data of HCC cells are from three independent experiments. **d** Correlation between mRNA expression indexes of TGFβ1 and PRG4 in HCC tumors (*N* = 78 patients). Tumor mRNA expression indexes of any specific marker used for plot generation represent the net tumor expression scores obtained by subtracting the non-tumor mRNA expression value from the corresponding value in matched tumor tissue in each patient. Data are expressed as the means ± SD (normalized to controls in **a** and **b**). GAPDH was used as housekeeping gene. *T* test (**a**–**c**; paired, one-tailed): *p < 0.05; **p < 0.01; ***p < 0.001. Log-rank test **d**.

To identify the cell types responsible for PRG4 production in HCC, we treated ex vivo cultured surgical tumor specimens, CAFs, and five HCC cell lines with TGFβ, the TGFβRI inhibitor LY2157299 (galunisertib), or both, for 48 hours, and then analyzed the PRG4 gene transcriptional response (Fig. 2). TGFβ significantly enhanced the expression of PRG4 mRNA in both HCC samples and CAFs (*p* < 0.05), whereas LY2157299 offset this effect (*p* < 0.05). Interestingly, LY2157299 also downregulated the expression of PRG4 in ex vivo samples in comparison with control (Fig. 2a, b). This may be due, at least partially, to the blockade of PRG4 expression evoked by residual endogenous TGFβ within the tissue. We also analyzed mRNA expression of αSMA, and three other high molecular weight PGs, CSPG4, HSPG2, and VCAN, in the same samples. The myofibroblast phenotype (detected by assessing αSMA expression) was emphasized in ex vivo samples (*p* < 0.05) and CAFs (non-significant) by TGFβ, but impaired only in CAFs by LY2157299 (*p* < 0.001). TGFβ also significantly increased the expression levels of HSPG2 and VCAN in HCC-cultured tissues (*p* < 0.05), and VCAN in CAFs (*p* < 0.01), but neither CSPG4 in CAFs and ex vivo samples, nor HSPG2 in CAFs, suggesting that CSPG4 expression is not regulated by TGFβ, and that HSPG2 may be produced in HCC under the control of this cytokine by cells other than CAFs. Overall, PRG4 transcription showed a higher degree of induction in response to a TGFβ stimulus, as compared to the other PGs examined. A representative immunophenotypic characterization of human HCC CAFs used in these experiments is shown in Supplementary Fig. 2.

We then screened HCC cells for PRG4 expression in response to TGFβ. Surprisingly, upon TGFβ1 treatment the accumulation of PRG4 transcript showed a marked increase in Hep3B and PLC/PRF/5 HCC cell lines, whereas this induction was absent or very low in HLE, HLF, and HLC19 cells (Fig. 2c, right). PRG4 mRNA response of CAFs from the four patients (Pt 1–4) used for panel b is shown for comparison (Fig. 2c, left). Analysis of microarray data in human HCC samples revealed a positive correlation between TGFB1 and PRG4 mRNA expression levels in a subset of patients with a better overall prognosis, whereas there was no correlation in the whole group of samples, or in those related to worse outcome (Fig. 2d). This evidence is consistent with the conclusion drawn by Coulouarn et al.^37^. According to these authors, HCCs with a more favorable prognosis are more likely to exhibit a TGFβ-related gene expression profile referred to as the early TGFβ signature, which also recurs in Hep3B and PLC/PRF/5 cell lines, as opposed to tumors with a worse prognosis, that are prone to display a late TGFβ signature, which in turn applies to HLE and HLF cell lines.

### Tumor PRG4 expression is correlated with a better prognosis in HCC patients with lower CD44 expression

Having found that TGFβ enhances the myofibroblast phenotype of HCC CAFs (higher αSMA expression) and promotes PRG4 expression by these cells, and some liver cancer cells, we aimed to determine a possible impact of the tumor tissue expression of PRG4 functionally associated TGFβ, αSMA, and the best known PRG4 receptor, CD44, on the survival of HCC patients.

**Fig. 3:**
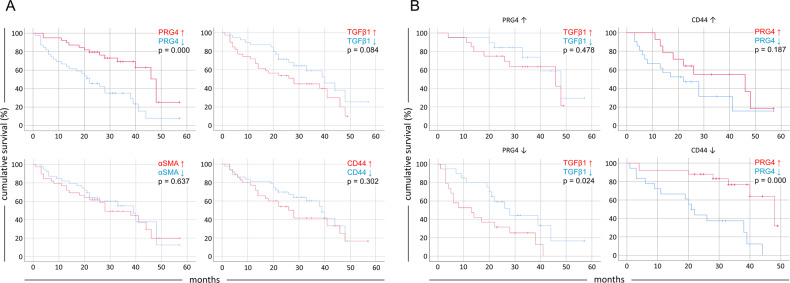
**Tumor expression of PRG4 is correlated with a better prognosis in HCC patients with concomitant lower tumor CD44 expression levels**. Kaplan–Meier curves showing the cumulative survival rate of 78 HCC cases. Patients are stratified into cohorts according to tumor mRNA expression indexes of each marker above or below the median values. Tumor mRNA expression indexes of any specific marker used for the generation of Kaplan–Meier curves represent the net tumor expression scores obtained by subtracting the non-tumor mRNA expression value from the corresponding value in matched tumor tissue in each patient. **a** Survival curves of patients expressing high or low mRNA levels of each marker. Note that the PRG4 chart in this panel is the corresponding chart of Fig. 1a, reused here for comparison purposes. **b** Survival curves of subsets of patients according to high or low PRG4 (left) and CD44 (right) tumor mRNA expression indexes.

To this end, we stratified the 78 HCC patients enrolled in the microarray analysis according to higher or lower tumor expression levels of PRG4 or of the other related genes and evaluated the overall survival rate related to each cohort. As for Fig. 1a, to determine the impact of expression of these genes on survival, net tumor mRNA expression indexes, obtained by normalizing tumor mRNA values to values of paired non-tumor specimen counterparts, were used. Kaplan–Meier curves with net tumor expression values were then plotted. Unlike PRG4, different expression levels of TGFβ, CD44, and αSMA are not associated with changes in clinical outcome (Fig. 3a). It is noteworthy that in patients with lower PRG4 levels, high TGFβ expression is related to a worse prognosis (Fig. 3b, left). This suggests that the tumor-suppressing role of TGFβ may be at least partially mediated by its ability to stimulate PRG4 expression, whereas a lack of this upregulation mechanism may result in unleashed TGFβ tumor-promoting actions. Even more interestingly, the putative effect of PRG4 as a “protective” factor was lost in those patients with higher CD44 expression, whereas a higher PRG4 expression was significantly correlated with better prognosis in patients with lower CD44 expression (*p* = 0.000) (Fig. 3b, right). This evidence suggests the possibility of CD44-independent PRG4 antitumor activities and/or a more intricate biochemical interplay between PRG4 and CD44 in vivo.

### Soluble rhPRG4 inhibits HCC cells migration without CD44 involvement

**Fig. 4:**
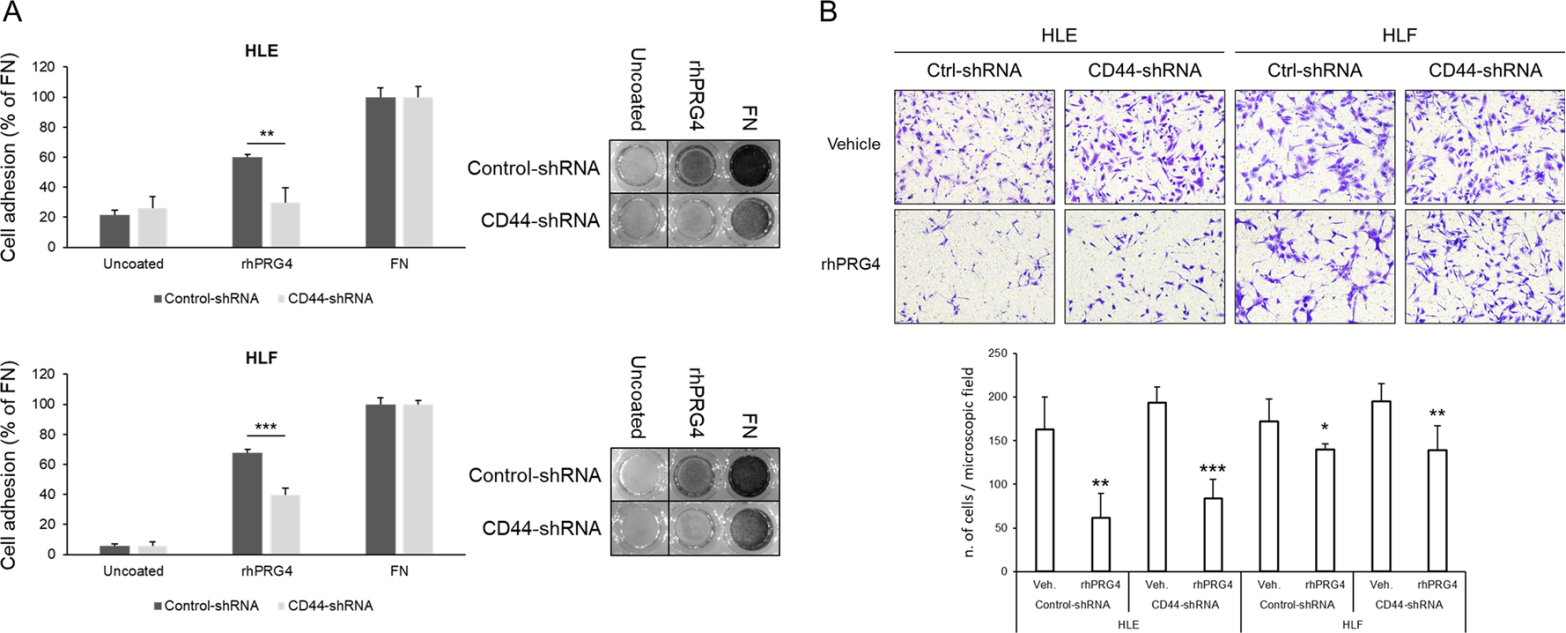
**rhPRG4 inhibits HCC cell migration on fibronectin without CD44 involvement**. **a** Cell adhesion assay. HLE or HLF cells were seeded on uncoated, or rhPRG4- or fibronectin (FN)-coated well surfaces, and allowed to attach and spread for 30 minutes. Data are the means ± SD of triplicates. **b** Transwell migration assay. Cells were seeded on the top of the transwell membrane, (previously coated with FN on the lower side) and allowed to migrate for 16 hours in the presence or not of soluble rhPRG4 (25 µg/ml) in the lower chamber. Data are the means ± SD of five randomly chosen microscopic fields. *T* test (unpaired, two-tailed): *p < 0.05; **p < 0.01; ***p < 0.001.

**Fig. 5:**
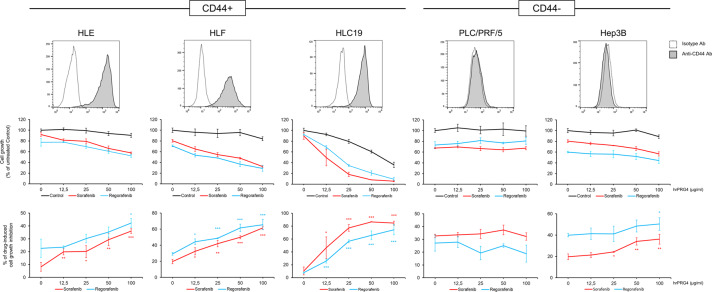
**rhPRG4 enhances sorafenib and regorafenib effectiveness in inhibiting cell proliferation, preferentially in high CD44-expressing HCC cells**. Cells were analyzed by flow cytometry to quantify CD44 expression (histograms), and tested for growth rate for 72 hours in the presence or absence of sorafenib, regorafenib at a fixed concentration (2.5 µM), and increasing rhPRG4 concentrations (0–100 µg/ml). Drug effectiveness (% of drug-induced cell growth inhibition) is calculated for each rhPRG4 concentration as the percentage of cell growth inhibition induced by combined drug + PRG4 with reference to control (rhPRG4 alone). Data are expressed as the means ± SD of triplicates. *T* test (unpaired, two-tailed): *p < 0.05; **p < 0.01; ***p < 0.001.

In an effort to explain how PRG4 might improve the overall survival of HCC patients, in particular those with CD44 expression levels below the median value (Figs. 1a and 3), we investigated whether full-length rhPRG4 affects cell migration as a key feature required for HCC cell aggressiveness, and whether CD44 expression is implicated. HLE and HLF are two highly CD44-positive invasive HCC cell lines (as will be shown later in Fig. 5). To assess whether rhPRG4 binds to CD44 we performed a cell adhesion assay using these cells upon stable silencing of CD44 expression (silencing efficiency of anti-CD44-shRNA sequences used is shown in Supplementary Fig. 1). CD44 knockdown cells showed a reduced capacity to adhere to surface-coated rhPRG4, as compared with control cells, and in reference to the adhesion capacity to FN (Fig. 4a). The migration of the same cells on FN was impaired by soluble rhPRG4 (25 µg/ml), but CD44 downregulation did not affect motility (Fig. 4b). This suggests that PRG4 produced in the HCC microenvironment can bind to CD44, but neither this interaction, nor the CD44 expression level seem to modulate the PRG4 effect on HCC cell migration. Thus, PRG4 might require alternative receptors, instead of CD44, to affect HCC cell migration. In addition to CD44, TLR2, and TLR4, which are typical surface receptors of immune cells, such as monocytes, macrophages, and dendritic cells, have also been recently discovered to bind PRG4^38,39^. After screening five HCC cell lines for the expression of these receptors, we found that both TLR2 and TLR4 are expressed also in HCC cells, although at higher levels in HLE, HLF, and HLC19, as compared with PLC/PRF/5 and Hep3B (Supplementary Fig. 4). Alternatively, PRG4 might have other antitumor activities, in addition to migration impairment, that are opposed by hyperactivated CD44 signaling.

### The CD44/PRG4 axis boosts sorafenib and regorafenib effectiveness on HCC cells

To explore the biological role of the PRG4/CD44 axis in further detail, we challenged HLE, HLF, and HLC19 HCC CD44-positive cells, and Hep3B and PLC/PRF/5 HCC CD44-negative cells with sorafenib and regorafenib in the presence/absence of rhPRG4 (Fig. 5). All HCC cells were grown for 72 hours in the presence/absence of rhPRG4 and sorafenib or regorafenib at a lower concentration (2.5 µM) than the IC50 (~5 µM) under the same experimental conditions. rhPRG4 alone did not markedly impair the cell proliferation but, when coupled with sorafenib or regorafenib, it strongly and synergistically improved their effectiveness on HLE, HLF, and HLC19 cells, at concentrations spanning from 12.5 to 100 µg/ml. The rhPRG4-drug synergistic effect was present during Hep3B proliferation, even if lower than that obtained with HLE, HLF and HLC19 cells, but only at the highest rhPRG4 concentrations. Instead, PLC/PRF/5 cells did not respond at all.

**Fig. 6:**
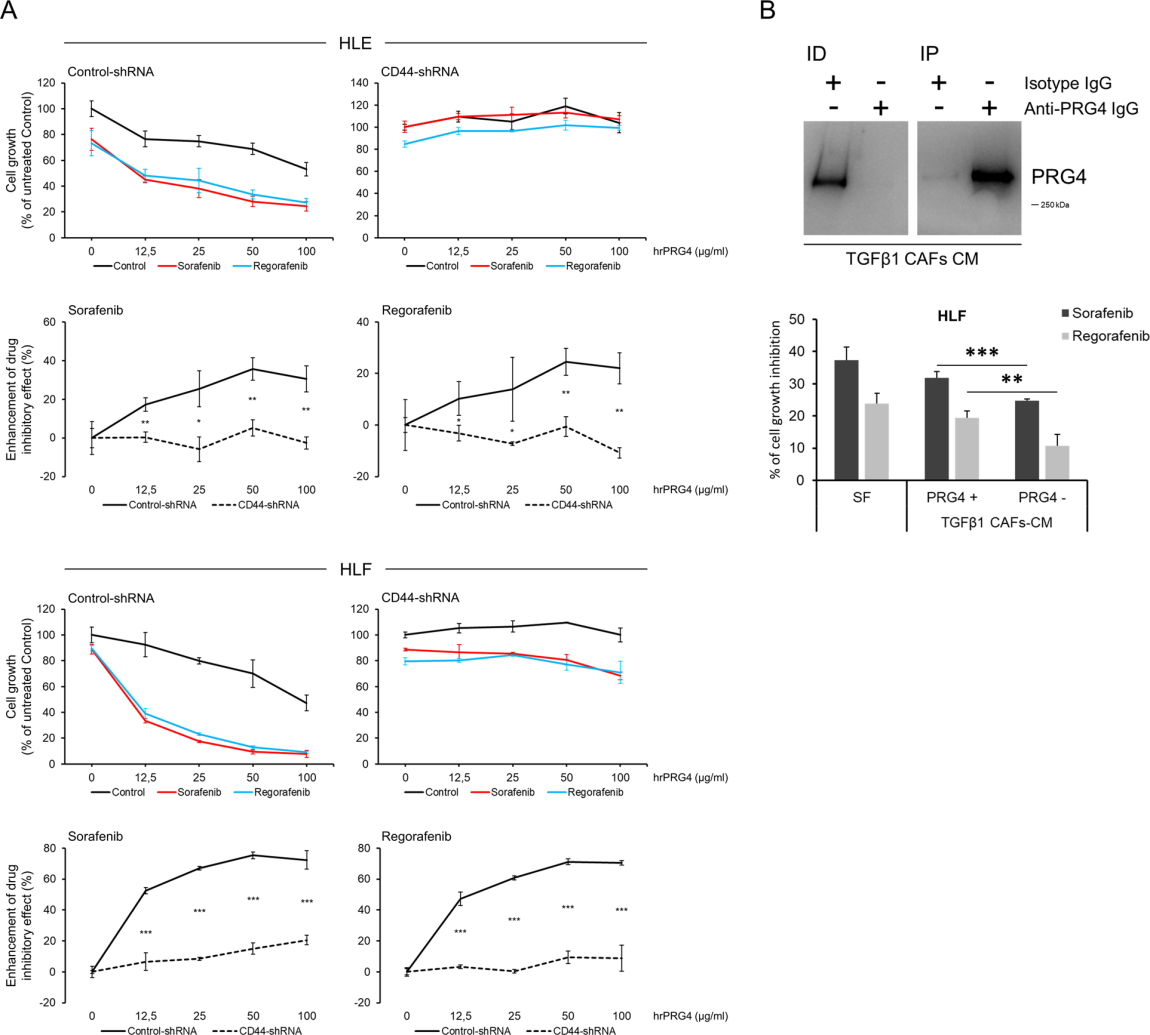
**CD44 silencing in HCC cells offsets the enhancement of drug effectiveness induced by rhPRG4**. **a** Cells were transduced via lentiviral infection and further selected for stable CD44-silencing. A 72-hour proliferation test was performed and the effect of increasing rhPRG4 concentrations (0–100 µg/ml) in enhancing the inhibitory action of sorafenib or regorafenib at a fixed concentration (2.5 µM) was plotted both for Control- and CD44-shRNA cells. The synergistic effect (enhancement of drug inhibitory effect) of combined drug + rhPRG4 is calculated for each rhPRG4 concentration as the percentage of cell growth inhibition by drug + PRG4, net of the effect of drug tested individually, with reference to control (rhPRG4 alone). **b** HCC CAFs were stimulated for 48 hours with TGFβ1 and further incubated for 48 hours (without TGFβ1) in serum-free conditions to allow enrichment of the conditioned medium (CM). The CM was then concentrated and PRG4-depleted or not. Western blot showing PRG4 depletion from CM of TGFβ1-treated CAFs. ID = immunodepleted TGFβ1-treated CAFs-CM using isotype or anti- PRG4 antibody; IP = immunoprecipitated PRG4 from TGFβ1-treated CAFs-CM using isotype or anti-PRG4 antibody. Whole blot scan is shown in Supplementary Fig. 5B. **b** Effect of TGFβ1-treated CAFs-CM PRG4 depleted/not depleted on sorafenib and regorafenib inhibitory action against HLF cell proliferation; 20 µg/ml of CM proteins were used in a 72-hour growth test in the presence of 1% FBS. Data are the means ± SD of triplicates. *T* test (unpaired, two-tailed): *p < 0.05; **p < 0.01; ***p < 0.001.

To test the hypothesis that CD44/PRG4 interaction is required to modulate drug-sensitivity, we used a CD44 loss of function approach based on stable shRNA-mediated silencing of CD44 expression in HCC cells. Control-shRNA and CD44-shRNA HLE and HLF cells were tested in a 72-hour proliferation assay in the presence/absence of rhPRG4 (at concentrations ranging from 0 to 100 µg/ml), and without or with sorafenib or regorafenib (at the fixed concentration of 2.5 µM) (Fig. 6a). The synergistic effect of rhPRG4 and each drug in slowing down cell proliferation was significantly reduced in CD44-silenced cells (*p* < 0.05 to *p* < 0.001 depending on rhPRG4 concentration), as compared with control cells, thus suggesting that PRG4/CD44 interaction is a requisite for PRG4 to enhance antiproliferative drug activity in vitro. The reduced drug effectiveness in rhPRG4-exposed CD44-silenced cells is consistent with the lesser sensitivity to rhPRG4 of drug-exposed low CD44-expressing HCC cell lines PLC/PRF/5 and Hep3B (see Fig. 5). Silencing efficiency of anti-CD44-shRNA sequences used is shown in Supplementary Fig. 1.

### PRG4 secreted in conditioned medium of TGFβ-stimulated CAFs increases sorafenib and regorafenib effectiveness

**Fig. 7:**
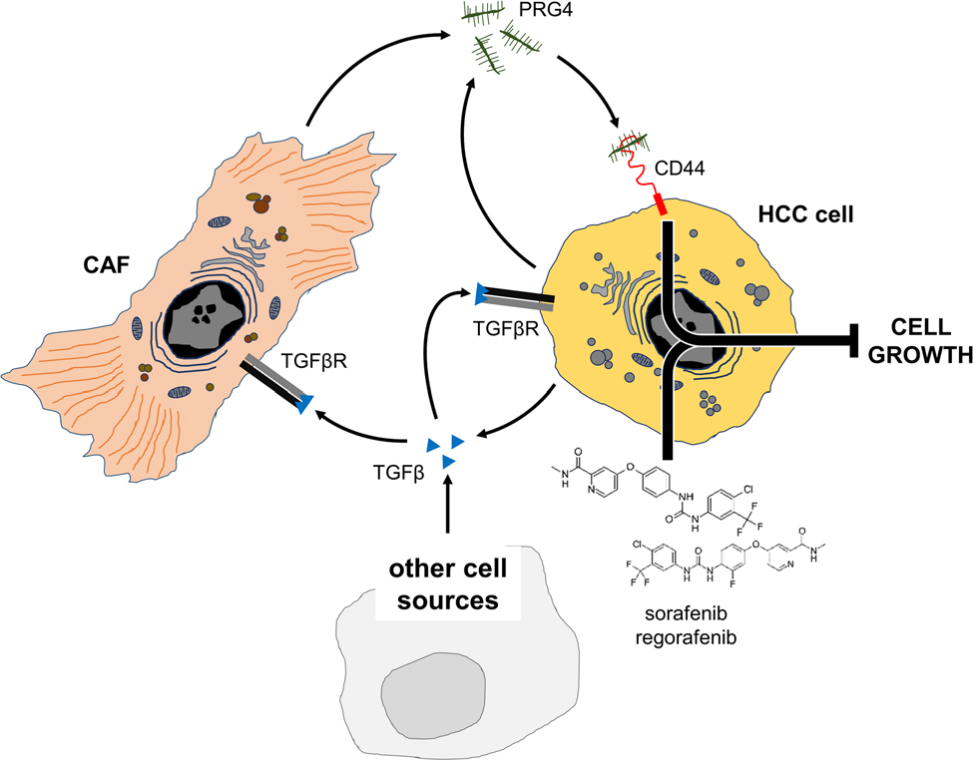
Schematic diagram of the findings of the study. Cartoon depicting the interactive functional network involving HCC cells, CAFs, CD44, and PRG4, and the enhancement of sorafenib and regorafenib antiproliferative effect by PRG4/CD44 axis. TGFβ induces the expression/secretion of PRG4 by cancer cells and CAFs. The binding of PRG4 to CD44 on HCC cells is required for the occurrence of the synergistic effect of combined PRG4 and sorafenib/regorafenib in hindering cell proliferation.

To further corroborate the evidence of PRG4-drug synergistic interaction, we reproduced in vitro the biological process that resembles a more physiological situation, whereby TGFβ-stimulated CAFs secrete PRG4, that, in turn, improves the drug effectiveness of both sorafenib and regorafenib on HCC cells. CAFs were incubated in the presence of TGFβ, in complete medium for 48 hours, and in starving condition (serum-free) for additional 48 hours (without TGFβ) to allow accumulation of secreted proteins. The conditioned medium was then collected, incubated with isotype, or anti-PRG4 antibody, assayed for protein concentration, and used to test the growth capacity of HCC cells in the presence of 1.5 µM sorafenib and regorafenib and/or 20 µg/ml (concentration of total proteins) of PRG4-depleted or not-depleted conditioned medium (CM) from TGFβ-stimulated CAFs. The conditioned medium from TGFβ-stimulated CAFs significantly (*p* < 0.01) increased sorafenib and regorafenib effectiveness on HLF cells, as compared with PRG4-depleted conditioned medium (Fig. 6b). This suggests that CAFs-secreted PRG4 enhances the effectiveness of sorafenib and regorafenib. The overall mechanism is summarized in Fig. 7.

## Discussion

In the present study, we provide evidence that the ECM component PRG4 is expressed in the HCC, with a preferential localization in the stromal milieu. According to our data, HCC CAFs, and a subset of HCC cancerous hepatocytes with less-invasive properties, secrete PRG4 upon TGFβ stimulation, and thus are probably a major source of this PG. The expression/secretion of PRG4 in response to TGFβ was previously reported, but limited to synovial fibroblasts and chondrocytes^40,41^. Most importantly, we demonstrate that, in CD44 overexpressing HCC cell lines, the CD44–PRG4 interaction leads to an increased sensitivity to the antiproliferative action of the multi-kinase inhibitors sorafenib and regorafenib in vitro. CD44 was previously identified by Al-Sharif and colleagues as a PRG4 receptor. By binding to CD44, PRG4 was shown to interfere with the intracellular signals downstream of this receptor, and thereby suppresses the proliferation of synoviocytes induced by proinflammatory cytokines^42^. Al-Sharif et al. found that PRG4 can compete with hyaluronan for binding to CD44, and then attenuates the growth-supporting signaling function of this receptor in rheumatoid arthritis fibroblast-like synoviocytes stimulated with interleukin-1β or tumor necrosis factor alpha^42^. Until a few years ago, PRG4 functions were predominantly known outside the cancer context. The function of this PG has been described in chondro-articular tissues. Studies using PRG4-knockout mice have determined that it protects cartilage surfaces against friction-induced joint deterioration, through a boundary-lubricating activity^28,43–45^. Based on this evidence PRG4 was recently shown to be a promising curative agent when exogenously administered to reverse osteoarthritic-related manifestations^33,35^. Nahon et al. showed that the absence of PRG4 increases the susceptibility to atherosclerosis in two hyperlipidemic mouse models already predisposed to atherosclerosis development, namely apolipoprotein E knockout (ApoE KO) mice and low-density lipoprotein receptor knockout (Ldlr KO) mice^46^. Reduced expression of TGFβ signaling is associated with age-related osteoarthritis in humans. In mice with defective TGFβ signaling, that effectively recapitulate human osteoarthritis, PRG4 has proven effective to prevent the onset of this disease, owing to its function as joints lubricant^34^. Besides in the synovium of cartilage sites of the joints, PRG4 was found to be expressed in some other tissues, including lung, heart and liver, although the evidence provided remains limited to the related mRNA^47–49^. Conversely, we found both PRG4 mRNA and protein expression in normal liver and HCC tumoral and surrounding peritumoral tissue. Moreover, we also demonstrate that PRG4 mRNA expression is positively correlated to longer survival in a cohort of 78 patients followed up over 5 years^50^. In in vitro assays, we also demonstrate that CD44 increases HCC cell adhesion to coated PRG4 but that migration on FN is inhibited by soluble rhPRG4 without the involvement of CD44. These data partially contribute to the interpretation of the clinical data, even if they are not fully consistent with the finding by Sarkar et al. These authors demonstrated that rhPRG4 is able to offset TGFβ-induced enhancement of migration and invasion of breast cancer cell line MDA-MB231, but this capacity requires rhPRG4 to interact with CD44 to interfere with its pro-invasive downstream signaling. In addition, they found a decreased expression of CD44 following binding to rhPRG4^23^.

The emerging antitumor role of PRG4 may be in apparent contradiction with the evidence that it is upregulated by TGFβ, when this cytokine is considered only as a tumor supporter. However, TGFβ is believed to play a dual, conflictual role in early and late cancer stages. Indeed, the knowledge accumulated so far suggests that TGFβ signaling behaves as a molecular switch, being cytostatic/pro-apoptotic in the initial development steps of solid malignancies, such as HCC, but turning into a metastasis-favoring factor in advanced phases^51–54^. More specifically, TGFβ signaling can affect CD44 expression/activation, and was shown to rely on CD44 functions to promote cancerous invasion. Ghatak et al.^55^. demonstrated that TGFβ upregulates the expression of the CD44 cancer-related CD44V6 isoform through EGR1-mediated AP-1 (activator protein-1) activation in pulmonary fibroblasts. Nevertheless, other studies have clearly highlighted the capacity of TGFβ to restrain the pro-tumorigenic potential of HCC cells, via inducing the expression of tumor suppressors genes, such as LATS1, or DNA damage repair proteins (ATM, BRCA1, and FANCF)^56,57^. A further layer of complexity of the TGFβ role in HCC is suggested by the positive correlation between TGFβ and poor survival, observed only in the patients’ subset expressing low PRG4 levels (see Fig. 3b, left).

We propose that the synergistic antiproliferative effect arising from a combination of rhPRG4 and sorafenib or regorafenib may result in a significantly improved therapeutic benefit for HCC patients. Our data show that CD44 expression is required for PRG4 to efficiently synergize with these drugs in blocking HCC cell proliferation. This is supported by the evidence that the cells with low or no CD44 expression (owing to shRNA-mediated silencing or constitutive absence) are less sensitive to PRG4. These findings warrant further investigations to determine what components of CD44 signaling are critical in mediating the effects of PRG4 in synergy with drugs to slow down HCC cell growth. As demonstrated by Alquraini et al. rhPRG4 can inhibit NFκB p50/p65 nuclear translocation in osteoarthritis synoviocytes and proliferation of these cells in a CD44-dependent manner^58^. Wu et al. showed that inhibition of NF-kappaB activity strongly sensitizes Hep3B cells to sorafenib-induced cell death^59^. Regorafenib was reported to inhibit NF-kB activity in various cancer cell lines, including HCC cells^60,61^. Therefore, we hypothesize that NF-kB may be a critical hub in the signaling mechanism that mediates the synergistic effect of PRG4 and drugs.

The limited, or absent capacity of rhPRG4 to slow down HCC cell proliferation in vitro is in apparent contrast with the strong positive correlation we observed between PRG4 expression and patients’ overall survival rates. A possible explanation may be possible not yet elucidated effects of this PG on other cell components of tumors, except for cancerous cells, that, in turn, might exert tumor-limiting activities. For example, PRG4 may contribute to restrain antitumor CD4+ or CD8+ T lymphocytes within the tumor by binding to CD44 expressed on these cells. Alternatively, by binding to CD44 or other receptors, PRG4 might play a role in sensitizing HCC cells to a variety of endogenous stressors. The positive correlation observed between PRG4 level and life expectancy only in patients with low CD44 expression might reflect a failure of PRG4 to saturate CD44 binding site/s, when this receptor is upregulated above a certain threshold, making it unable to overcome tumor-promoting functions of CD44 signaling. Another explanation may be that potential CD44-independent in vivo effects of PRG4 might be opposed or overwhelmed by CD44 pro-malignant pathway when this receptor is significantly overexpressed.

Overall, our results propose a novel interventional framework, where CD44 inhibition may be coupled with rhPRG4 administration in high CD44 expression HCCs. Moreover, the potency of sorafenib and regorafenib may be greatly enhanced by a synergistic administration with rhPRG4. In addition, using a recombinant protein that mimics the molecular structure of a naturally produced compound should theoretically reduce the risk of side effects, as compared with administering exogenous synthetic pharmacologic agents. Al-Sharif et al.^42^ demonstrated that the efficiency of PRG4 binding to CD44 can be significantly increased after the removal of sialic acid and *O*-glycosylation. Therefore, potentially detrimental off-target effects of PRG4 could be minimized by using lower concentrations of this processed form. Evaluation of possible negative effects of exogenous PRG4 administration in experimental animal models will be necessary. More specifically, PRG4 delivery via local or systemic administration (i.e., intraperitoneal or intravascular) must be performed to assess its tolerability in vivo.

We suggest that PRG4 has the potential to act as an antitumor agent, but also to improve the effectiveness of drugs such as sorafenib and regorafenib. Further insights into the molecular mechanisms of PRG4 synthesis in the stromal HCC microenvironment, as well as its functional features, possibly gained through identifying novel PRG4-coupling factors, alternative receptors, or partners of interactions, may prove valuable for the purpose of designing more effective pharmacologic tools for HCC treatment.

## Supplementary information

Supplementary figure and table legends

Efficiency of CD44 silencing by stably expressed specific shRNAs.

Immunophenotypic characterization of primary human HCC CAFs.

Morphologic and immunophenotypic characterization of HLC19 primary HCC cells.

Flow cytometry analysis of TLR2 and TLR4 expression in HCC cell lines.

Whole blot scans of Fig. 1B (A), 6B (B), and Supplementary Fig. 1 (C).

Antibodies used.

List of Real-Time Polymerase Chain Reaction (qPCR) primers.
