# Supplementary material for: Proteoglycan-4 is correlated with longer survival in HCC patients and enhances sorafenib and regorafenib effectiveness via CD44 in vitro
Source: Cell Death Dis. 2020 Nov 16;11(11):984. doi: 10.1038/s41419-020-03180-8 (PMC7669886; doi:10.1038/s41419-020-03180-8)
Supplement: Supplementary file 1 — Supplementary figure and table legends [file 41419_2020_3180_MOESM1_ESM.docx]

Suppl. Fig. 1 Efficiency of CD44 silencing by stably expressed specific shRNAs. (*left*) Analysis (qPCR) of silencing efficiency of four CD44-shRNA sequences (A to D), compared to Control-shRNA sequence (V) in HLE and HLF cells following lentiviral transduction and puromycin selection for stable silencing of CD44 expression. GAPDH was used as housekeeping gene. qPCR data are normalized to Control-shRNA. (*right*) Western blot (WB) showing the silencing efficiency of CD44-shRNA sequence B in HLF and HLE cells. GAPDH was used as housekeeping gene. V and B sequences were used in all subsequent experiments involving CD44 silencing. Whole blot scans are shown in Supplementary Fig. 5C.

Suppl. Fig. 2 Immunophenotypic characterization of primary human HCC CAFs. Immunofluorescence and flow cytometry analysis (scale bar: 50 µm).

Suppl. Fig. 3 Morphologic and immunophenotypic characterization of HLC19 primary HCC cells. The expression of common epithelial-, mesenchymal-, and stemness-related markers is shown. Scale bar: 100 µm.

Suppl. Fig. 4 Flow cytometry analysis of TLR2 and TLR4 expression in HCC cell lines.

Suppl. Fig. 5 Whole blot scans of Fig. 1B (A), 6B (B), and Supplementary Fig. 1 (C).

Suppl. Table 1 Antibodies used.

Suppl. Table 2 List of Real-Time Polymerase Chain Reaction (qPCR) primers.
