# Supplementary material for: Proteoglycan-4 is correlated with longer survival in HCC patients and enhances sorafenib and regorafenib effectiveness via CD44 in vitro
Source: Cell Death Dis. 2020 Nov 16;11(11):984. doi: 10.1038/s41419-020-03180-8 (PMC7669886; doi:10.1038/s41419-020-03180-8)
Supplement: Supplementary file 7 — Antibodies used. [file 41419_2020_3180_MOESM7_ESM.docx]

**Supplementary Table 1.** Antibodies used

| **Antigen** | **Conjugated fluorophore** | **Clone** | **Catalog #** | **Brand** |
| --- | --- | --- | --- | --- |
| PRG4 |  | 9G3 | MABT401 | Merck |
| PRG4 |  |  | PA3-118 | Thermo Fisher Scientific |
| α-SMA |  | D4K9N | 19245 | Cell Signaling Technology |
| Vimentin |  | VI-10 | ab20346 | abcam |
| CD13 | FITC | WM-47 | FCMAB180F | Merck |
| CD151 | FITC | 11G5a | MA1-81266 | Thermo Fisher Scientific |
| OV-6 |  | OV-6 | MAB2020 | R&D Systems |
| CD133 | FITC | 13A4 | 11-1331-82 | Thermo Fisher Scientific |
| EpCAM | FITC | G8.8 | 11-5791-82 | Thermo Fisher Scientific |
| AFP | AF488 | AFP3 | 53-6583-82 | Thermo Fisher Scientific |
| No target | AF488 | MOPC-21 | MA5-18167 | Thermo Fisher Scientific |
| No target | FITC |  | GM4992 | Thermo Fisher Scientific |
| CD44 | FITC | IM7 | 11-0441-82 | Thermo Fisher Scientific |
| CD90 | FITC | eBio5E10 (5E10) | 11-0909-42 | Thermo Fisher Scientific |
| E-cadherin | FITC | 67A4 | A15757 | Thermo Fisher Scientific |
| Rabbit IgG | AF594 |  | A32740 | Thermo Fisher Scientific |
| Mouse IgG | AF488 |  | A-11001 | Thermo Fisher Scientific |
| FAK (pY397) |  | 14/FAK(Y397) | 611722 | BD |
| CK19 |  | EP1580Y | ab52625 | abcam |
| N-cadherin |  |  | ab76057 | abcam |
| TLR2 |  |  | BS-1019R | Thermo Fisher Scientific |
| TLR4 |  |  | BS-20595R | Thermo Fisher Scientific |
