# Supplementary material for: Proteoglycan-4 is correlated with longer survival in HCC patients and enhances sorafenib and regorafenib effectiveness via CD44 in vitro
Source: Cell Death Dis. 2020 Nov 16;11(11):984. doi: 10.1038/s41419-020-03180-8 (PMC7669886; doi:10.1038/s41419-020-03180-8)
Supplement: Supplementary file 8 — List of Real-Time Polymerase Chain Reaction (qPCR) primers. [file 41419_2020_3180_MOESM8_ESM.docx]

**Supplementary Table 2.** Real-Time Polymerase Chain Reaction Primers

| Gene | Species | Primer sequence (5′ to 3′) |
| --- | --- | --- |
| *ACTA2* | *Homo sapiens* | Forward: GGAATGGGACAAAAAGACAGCTA |
|  |  | Reverse: CGGGTACTTCAGGGTCAGGAT |
| *CSPG4* | *Homo sapiens* | Forward: CACGGATGCCACCCTACAAG |
|  |  | Reverse: TCCTGGGCTGCCTCCAG |
| *HSPG2* | *Homo sapiens* | Forward: TGAGTCCTTCTACTGGCAGC |
|  |  | Reverse: GTTGTTGCCCGTGATCTGC |
| *PRG4* | *Homo sapiens* | Forward: TCCCATGCTTTCCGATGAGAC |
|  |  | Reverse: ATTCTGCGAGCTGGAGATGG |
| *VCAN* | *Homo sapiens* | Forward: AGTGATGCGGGTCTTTACCG |
|  |  | Reverse: ACACAACCCCATCCACAGTC |
| *GAPDH* | *Homo sapiens* | Forward: CACCATCTTCCAGGAGCGAG |
|  |  | Reverse: GACTCCACGACGTACTCAGC |
| *CD44* | *Homo sapiens* | Forward: TGGAGAAAAATGGTCGCTACAG |
|  |  | Reverse: GGGCAAGGTGCTATTGAAAGC |
